# From Gender Threat to Farsightedness: How Women’s Perceived Intergroup Threat Shapes Their Long-Term Orientation

**DOI:** 10.3390/bs15111542

**Published:** 2025-11-13

**Authors:** Yongheng Shi, Yufang Zhao, Xingyang Ma, Shasha Chen

**Affiliations:** Faculty of Psychology, Southwest University, Chongqing 400175, China; swuaheng@email.swu.edu.cn (Y.S.); xingyang.ma@research.uwa.edu.au (X.M.); chenss1223@email.swu.edu.cn (S.C.)

**Keywords:** gender intergroup threat, intertemporal decision-making, delay discounting, cognitive appraisal, future orientation

## Abstract

Women experience realistic and symbolic gender intergroup threats across diverse social contexts, which can profoundly influence their decision-making processes. Drawing on intergroup threat theory, this research investigated how perceived gender intergroup threats affect women’s intertemporal choice behavior and examined cognitive appraisal as a potential mediating mechanism. Study 1 (*N* = 281) found a negative correlation between gender intergroup threat perception and delay discounting through questionnaires. Study 2 (*N* = 154) experimentally manipulated threat perception and demonstrated that both realistic and symbolic gender threats enhanced consideration of future consequences, with cognitive appraisal serving as a complete mediator of these effects. Study 3 (*N* = 120) employed a recall paradigm, providing convergent evidence that heightened realistic threat perception and associated threat appraisal increased preferences for delayed, long-term outcomes. These findings suggest that perceived gender intergroup threats promote future-oriented decision-making among women, potentially as an adaptive strategy to manage threat-related risks, and the mediating role of cognitive appraisal further elucidates the psychological mechanisms underlying this behavioral shift. This research advances the theoretical understanding of how intergroup threat dynamics shape women’s economic behavior and extends knowledge of gender threat interactions in decision-making contexts.

## 1. Introduction

Despite advances in gender equality, women continuously face systemic disadvantages across diverse social contexts (Ryan & Morgenroth, 2024). Worldwide, women hold only 30% of managerial positions, dedicate two and a half times more hours to unpaid care work compared to men, and comprise just 27.2% of national parliament members (Sustainable Development Goals Report 2025, United Nations Department of Economic and Social Affairs [UN DESA], 2025). In China, gender parity has gradually improved in recent years, yet the country remains ranked 103rd globally, with persistent gaps in political and economic participation (Global Gender Gap Report 2025, World Economic Forum, 2025). Beyond these overt inequities, pervasive forms of sexism—ranging from benevolent and modern to implicit—continue to hinder women’s educational and career advancement (Off et al., 2022; Kahn et al., 2021; Koudenburg et al., 2021).

Embedded within these structural inequalities, gender intergroup threat, as conceptualised in Intergroup Threat Theory, refers to the enduring perception and subjective experience of potential harm or devaluation to one’s gender group as a result of intergroup dynamics (C. W. Stephan et al., 2000). While substantial evidence demonstrates that perceived gender intergroup threats shape outgroup attitudes and behavior (Hopkins-Doyle et al., 2024; Sarter et al., 2024), far less is known about how members of disadvantaged gender groups themselves appraise and adapt to such threats.

Notably, gender intergroup threat does not manifest uniformly across groups. For men, such threats are often experienced as perceived challenges to extant privileges, status hierarchies, or gendered entitlements. For example, gender equity initiatives may be interpreted as threats to dominance, triggering physiological stress responses (Domen et al., 2022), increasing resistance to gender diversity efforts (Jones et al., 2022), and opposing feminist movements (Rivera-Rodriguez et al., 2022). Conversely, women’s experiences are shaped by their structurally disadvantaged positions (W. G. Stephan et al., 2009), encompassing both realistic (e.g., inequitable access to political power, economic resources, and personal safety) and symbolic threats (e.g., negative stereotypes and restrictive norms). These differing threat experiences underscore the importance of investigating unique adaptation mechanisms, especially among women.

From the perspective of the psychological shift model, environmental threat cues can induce changes in cognition and behaviour that promote adaptive responses (Sheehy-Skeffington, 2020). Prior work has revealed that individuals with low socioeconomic status often demonstrate “short-sighted” behaviour, forgoing long-term investments in favor of immediate gains when confronted by threat (Du et al., 2022; Pepper & Nettle, 2017). Analogously, structural barriers and discrimination affecting women may alter their orientation toward the future and shape long-term goal strategies, influencing education, career, and health decisions (Steinberg et al., 2009). For instance, women encountering gender-biased hiring practices may seek employment that offers stability and immediate rewards, even at the cost of reduced long-term prospects (Peters et al., 2013), or alternatively, may choose to invest in further qualifications to address enduring inequities (Diekman et al., 2011).

A key process underpinning these adaptive behaviours is intertemporal decision-making—the evaluation of trade-offs between immediate and delayed outcomes (Mischel et al., 1989). The delay discounting rate provides a behavioral measure of the extent to which future rewards are devalued (Green & Myerson, 2004), while the Consideration of Future Consequences (CFC) captures dispositional differences in prioritizing long-term outcomes (Strathman et al., 1994; Joireman et al., 2008), both of which can reflect individual’s future-orientation (Vonasch & Sjåstad, 2021). While acute threats generally elicit stress (Scheepers & Knight, 2020), negative emotions (Jiang et al., 2022), and a bias towards immediate rewards (Sharma et al., 2023; Simon et al., 2021), emerging evidence suggests that chronic, pervasive threats like those related to persistent gender inequality may paradoxically foster future-oriented, adaptive strategies as a means to cope and increase long-term security (Van Laar et al., 2019). In such cases, ongoing threats may also activate group-level concerns, encouraging collective action and long-term planning to secure resource stability (Rutchick et al., 2008; Stürmer & Simon, 2004).

The cognitive appraisal theory offers a promising framework for unpacking these nuanced effects (Alter et al., 2010). According to this theory, individuals evaluate stressful circumstances in terms of coping resources and demands, leading to challenge appraisal when resources are perceived as sufficient and threat appraisal when demands are seen as overwhelming (Blascovich & Tomaka, 1996; Lazarus & Folkman, 1984). These appraisals differentially influence emotional and behavioral outcomes: threat appraisals are linked to heightened stress and impaired performance, whereas challenge appraisals often promote adaptive responses (Tomaka et al., 1997; Blascovich et al., 2004). Notably, in the context of chronic intergroup threats, even challenge appraisals may have complex or paradoxical effects on executive functioning (Li & Zhao, 2015). For women exposed to persistent gender intergroup threat, threat appraisal may heighten vigilance and motivate strategic, long-term coping behaviour (Blascovich & Mendes, 2000), reflected in a greater prioritization of future-oriented choices.

Existing research highlights the adverse effects of gender discrimination on women’s well-being and achievement (Hackett et al., 2019, 2024; Schmitt et al., 2014; Vigod & Rochon, 2020), but few studies have examined its influence on decision-making behavior, particularly in non-Western contexts (Steinberg et al., 2009; Diekman et al., 2011). Moreover, research on gender intergroup threat has predominantly focused on men’s resistance to equality initiatives and loss of status (e.g., Domen et al., 2022; Jones et al., 2022), whereas little is known about how these dynamics affect women’s adaptive responses in the face of structural disadvantage (W. G. Stephan et al., 2009). To help address this imbalance, the current study investigates how Chinese female university students perceive and respond to gender-based intergroup threat, expanding the discussion to include decision-making processes. On the other hand, although cognitive appraisal mechanisms have been well-documented in the stereotype threat and acute stress literature (e.g., Berjot et al., 2011; Blascovich et al., 2004; Turner et al., 2024), their role in how women react to broader, ongoing intergroup threats within gender hierarchies remains scarce (Scheepers & Knight, 2020; Li & Zhao, 2015). Likewise, studies of intertemporal decision-making have also mainly concentrated on individual and situational factors, such as socioeconomic status and personality (Du et al., 2022; Van Laar et al., 2019), with minimal attention paid to the impact of group-level threats. By integrating perspectives from cognitive appraisal and decision-making theories, this study seeks to systematically evaluate the effects of persistent gender-based threats at the group level, thereby filling a critical gap shared by both domains and contributing to a more comprehensive understanding of women’s psychological adaptation.

The present research aims to elucidate the processes by which perceived gender intergroup threats shape women’s intertemporal decision-making, with particular emphasis on the mediating role of cognitive appraisal. Employing both questionnaire and experimental methodologies, we examine (1) the association between perceived gender intergroup threat and intertemporal choice among women, and (2) whether cognitive appraisal mediates this relationship. By advancing knowledge of the psychological mechanisms underlying adaptive decision-making under gender threat, this research seeks to inform effective interventions and policy development to support women’s empowerment and promote gender equity.

## 2. Study 1

### 2.1. Materials and Methods

#### 2.1.1. Participants

A total of 305 female college students were recruited from universities in mainland China via convenience sampling, reflecting a population particularly sensitive to gender-related threats amid social change (Lv et al., 2023; UN Women, 2023). Participants completed an online questionnaire using the Wenjuanxing platform (www.wjx.com, accessed on 10 November 2023) and received compensation for their involvement. After excluding 24 responses for incompleteness, duplicate submissions, or implausibly short completion times, the final sample included 281 women (*M*_age_ = 20.96, *SD* = 2.54), resulting in an effective response rate of 92.13%. Post hoc sensitivity analysis using G*Power 3.1 (Faul et al., 2007) indicated that this sample size was sufficient to detect a small effect size (*d* = 0.17) at *α* = 0.05 with power of 0.80.

#### 2.1.2. Measures

Unless otherwise specified, all items were rated on a 7-point Likert scale ranging from 1 (strongly disagree) to 7 (strongly agree).

Gender Intergroup Threat Perception: The participants’ perceptions of intergroup threats were assessed using the Gender Intergroup Threat Scale developed by C. W. Stephan et al. (2000). This scale comprises nine items that measure perceived threats posed by men to women’s economic, political, and personal security (e.g., “Men have too much political power” and “Many women live in fear of men’s aggression”), and ten items that assess perceptions of threats to women’s values and beliefs by men (e.g., “Men should not regard women as sex objects” and “Men put too little emphasis on family values”). Responses were averaged separately for realistic (*α* = 0.90) and symbolic threats (*α* = 0.84), with higher scores indicating stronger perceived threat.

Gender Identity: Gender identity strength was measured using the Private and Identity subscales of the Collective Self-Esteem Scale (Luhtanen & Crocker, 1992; Jones et al., 2022). This scale comprises eight items (e.g., “I feel good about being female” and “Being female is an important reflection of who I am”). Items were averaged to compute an overall gender identity score (*α* = 0.71), with higher scores reflecting stronger female ingroup identification.

Intertemporal Decision-making: Delay discounting was assessed using the 27-item Monetary Choice Questionnaire (MCQ-27; Kirby et al., 1999; Lv et al., 2023). Each item presented a choice between a smaller immediate reward (e.g., ¥25 today) and a larger delayed reward (e.g., ¥30 in 80 days). The monetary values ranged between ¥11 and ¥85, and the delays varied between 1 week and 186 days. The delay discounting rate (*k* value) was calculated using the scoring tool developed by Kaplan et al. (2016), with higher *k* values indicating stronger present-oriented preferences (i.e., greater delay discounting).

Subjective Social Class: Subjective social class was measured using the MacArthur Scale of Subjective Social Status (Adler et al., 2000). Participants viewed a picture of a labelled ladder to represent varying levels of income, education, and occupational status in China and responded to the following prompt: *“Where would you place your family on this ladder?”* Participants marked their perceived social standing on a 10-point scale, with higher numbers reflecting a higher subjective social status.

Objective Social Class: Objective social class was assessed using a five-item index that captured the parents’ educational attainment, occupational status, and annual family income (Chen et al., 2021). Each indicator was standardised and averaged to yield a composite score, with higher values indicating a higher objective social status.

### 2.2. Results

Harman’s one-factor test was used to assess the potential for common method bias. An exploratory factor analysis of all variables yielded 26 factors, with the first factor accounting for 30.28% of the total variance. Given that this value was below the conventional threshold of 40%, common method bias was not considered a major concern in this study.

Descriptive statistics and bivariate correlations among the study variables are presented in Table 1. Given the skewness in the distribution of the raw delay discounting rate (*k*), the natural logarithm of *k* was computed (denoted as *k*_0_) for all subsequent analyses (Jiang et al., 2022).

The correlation analyses indicated that gender realistic threat perception was significantly negatively correlated with the delay discounting rate (*r* = −0.18, *p* = 0.003); therefore, a higher perceived realistic threat was associated with a greater preference for delayed, larger rewards. Conversely, the correlation between gender symbolic threat perception and the delay discounting rate was not significant (*r* = −0.06, *p* = 0.336). Gender identity was significantly positively correlated with both realistic (*r* = 0.18, *p* = 0.003) and symbolic gender threat perceptions (*r* = 0.12, *p* = 0.038). In addition, the delay discounting rate was positively correlated with age (*r* = 0.14, *p* = 0.021) and subjective social status (*r* = 0.18, *p* = 0.002); therefore, these variables were included as covariates in all the regression analyses.

Hierarchical linear regression analyses were conducted to examine the unique contributions of realistic and symbolic gender threat perceptions on delay discounting rates (see Table 2). In Step 1, the age and subjective social status were entered as control variables. In Step 2, realistic and symbolic gender threat perceptions were simultaneously entered. The results indicated that, after adjusting for age and subjective social status, gender realistic threat perception was a significant negative predictor of delay discounting rate (*β* = −0.40, *p* = 0.004), whereas gender symbolic threat perception was not a significant predictor (*β* = 0.27, *p* = 0.138).

### 2.3. Discussion

Study 1 demonstrated that higher perceptions of realistic gender threats among women were associated with lower delay discounting rates, reflecting an increased propensity to select larger, future-oriented rewards over smaller, immediate ones. This finding suggests that, in contexts where women perceive tangible threats to their economic or physical well-being posed by men, they may adopt more future-oriented decision-making strategies. Notably, symbolic gender threats, related to values and beliefs, exhibited no significant relationship with intertemporal decision-making.

Building on these findings, Study 2 employed an experimental design to establish the causal effect of gender intergroup threat perception on intertemporal decision-making. Furthermore, given that cognitive appraisal processes are theorised to impact executive function, Study 2 examined the mediating role of cognitive appraisal in the relationship between gender intergroup threats and intertemporal decision-making.

## 3. Study 2

### 3.1. Methods

#### 3.1.1. Participants

A priori power analysis for a one-way ANOVA was conducted using G*Power 3.1 (Faul et al., 2007) based on an estimated effect size of *f* = 0.26 (Jones et al., 2022). The results indicated that a minimum sample size of 147 would provide 80% statistical power at *α* = 0.05. The final sample included 154 female college students from China between the ages of 18 and 24 years (*M*_age_ = 20.62, *SD* = 1.29).

#### 3.1.2. Procedure and Materials

Participants were randomly assigned to one of three experimental conditions: realistic threat (*n* = 55), symbolic threat (*n* = 54), or control (*n* = 45). The study sequence was as follows: (a) completion of the gender identity scale (Cronbach’s *α* = 0.77) to ensure female identity; (b) exposure to the gender intergroup threat priming materials with subsequent manipulation checks; (c) completion of the cognitive appraisal scale; and (d) completion of measures of intertemporal decision-making, including the Monetary Choice Questionnaire and the Consideration of Future Consequences Scale. The gender identity and monetary choice measures were consistent with those used in Study 1.

Gender Intergroup Threat Manipulation: Gender threats were primed using materials adapted from established intergroup threat paradigms (Rios et al., 2018), comprising text and images. The realistic threat group viewed content that emphasised salary gaps and promotion barriers for women in Chinese workplaces. The symbolic threat group received material emphasising the underestimation of women’s societal contributions and prevailing gender biases. The control group read a neutral passage about the Zhangjiajie National Forest Park.

Pre-testing was conducted with 20 female graduate students who rated the level of threat and perceived credibility of each material on 7-point scales. Both realistic (*M* = 5.20, *SD* = 1.01) and symbolic threats (*M* = 5.20, *SD* = 1.28) elicited a significantly greater perceived threat than the control condition (*M* = 1.15, *SD* = 0.37; *p*s < 0.001), confirming the effectiveness of the manipulations.

Cognitive Appraisal: Consistent with previous studies (Tomaka et al., 1997), cognitive appraisal was assessed using two items, each rated on a 7-point scale. Primary appraisal was measured using, “How threatening is the phenomenon described in the material to you?” Secondary appraisal was measured using, “How able are you to cope with the difficulties described in the material?” The composite index of cognitive appraisal was calculated as the ratio of primary to secondary appraisals. This index quantifies the degree to which perceived threats exceed one’s capacity to cope (Blascovich & Tomaka, 1996; Lazarus & Folkman, 1984).

Consideration of Future Consequences: Participants’ propensity to consider long-term versus immediate consequences was measured using the 12-item Consideration of Future Consequences Scale (Strathman et al., 1994), using a 7-point Likert scale, with higher scores reflecting a greater future orientation. An example item is “I am willing to sacrifice my current happiness and joy for future achievements”. The internal consistency for this scale in the current sample was acceptable (Cronbach’s *α* = 0.79).

### 3.2. Results

#### 3.2.1. Manipulation Check

To evaluate the effectiveness of threat manipulation, participants rated their threat-related emotional responses (e.g., threat, worry, anger, fear, and anxiety) using established items from a previous study (Wilson & Hugenberg, 2010). A one-way analysis of variance (ANOVA) revealed significant differences in threat emotions across the realistic threat, symbolic threat, and control conditions, *F*(2, 151) = 197.02, *p* < 0.001, $\eta_{p}^{2}$ = 0.72, 90% CI [0.66, 0.76]. Both the realistic (*M* = 3.37, *SD* = 0.69, *p* < 0.001) and symbolic threat groups (*M* = 3.32, *SD* = 0.75, *p* < 0.001) reported significantly higher threat emotion scores than the control group (*M* = 1.17, *SD* = 0.24), confirming the effectiveness of gender intergroup threat manipulations.

#### 3.2.2. Effect of Gender Intergroup Threat on Intertemporal Decision-Making

Intertemporal decision-making was assessed using the delay discounting rate and Consideration of Future Consequences (CFC) scores. The delay discounting rates were calculated using the same procedure as in Study 1.

**Figure 1:**
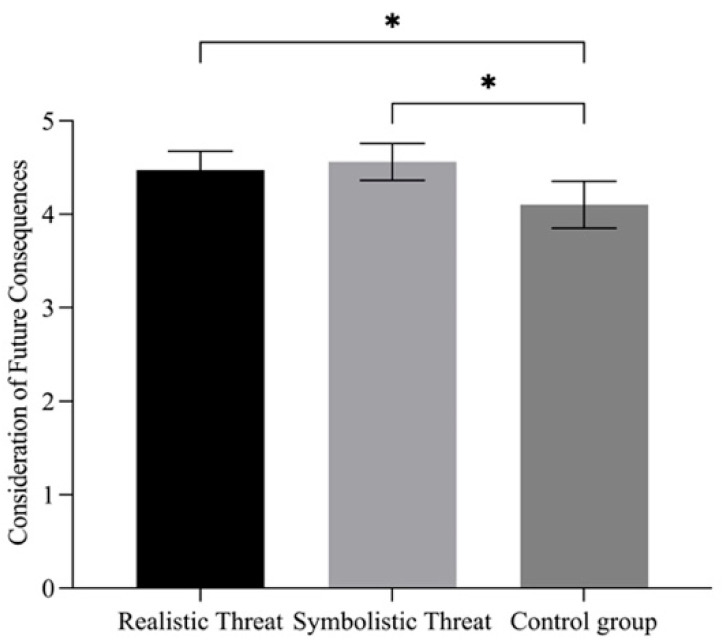
ANOVA results of Consideration of Future Consequences in Study 2. Note. * *p* < 0.05.

A one-way ANOVA indicated that delay discounting rates did not significantly differ among the three groups, *F*(2, 151) = 2.43, *p* = 0.092, $\eta_{p}^{2}$ = 0.03, 90% CI [0.00, 0.08]. However, a significant effect was observed for consideration of future consequences (see Figure 1), *F*(2, 151) = 4.73, *p* = 0.01, $\eta_{p}^{2}$ = 0.06, 90% CI [0.01, 0.12]; Post hoc pairwise comparisons were conducted using independent samples *t*-tests (with Bonferroni correction for multiple comparisons). Participants in both the realistic (*M* = 4.47, *SD* = 0.76, *t* = 2.38, *p* = 0.048, Cohen’s *d* = 0.48) and symbolic threat groups (*M* = 4.56, *SD* = 0.72, *t* = 2.93, *p* = 0.011, Cohen’s *d* = 0.59) scored significantly higher on future consideration than the control group (*M* = 4.10, *SD* = 0.84), suggesting that exposure to either form of gender intergroup threat enhanced participants’ future-oriented thinking.

#### 3.2.3. Mediating Role of Cognitive Appraisal

To test the potential mediating role of cognitive appraisal, gender intergroup threat was dummy coded, and the delay discounting rate (log-transformed *k* value; *k*_0_) and consideration of future consequences were entered as outcome variables. Cognitive appraisal was operationalised as the ratio of threat appraisal scores to challenge appraisal scores (Li & Zhao, 2015). Additionally, mediation analyses were conducted using PROCESS v4.2 (Model 4; bootstrapping, *n* = 5000, 95% CI; see Table 3).

**Figure 2:**
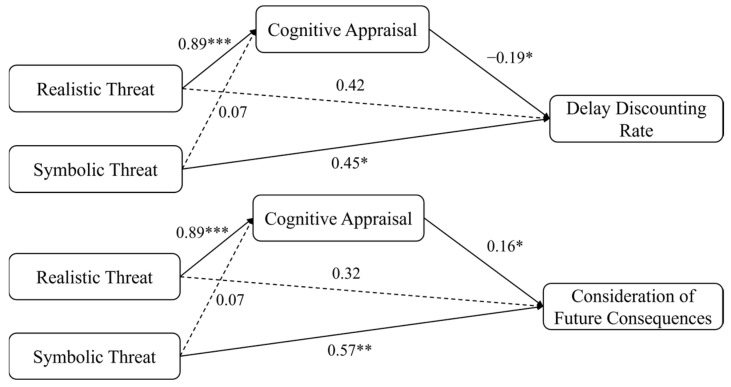
The Mediating Role of Cognitive Appraisal between Gender Intergroup Threats and Future-Orientation. Note. Path coefficients were standardised. * *p* < 0.05, ** *p* < 0.01, *** *p* < 0.001.

The results indicated that cognitive appraisal fully mediated the relationship between realistic threat and delay discounting rate (indirect effect = −0.284, 95% CI [−0.56, −0.05]), but not between symbolic threat and delay discounting rate (indirect effect = −0.022, 95% CI [−0.10, 0.07]). When considering CFC as the dependent variable, cognitive appraisal fully mediated the effect of realistic threats (indirect effect = 0.114, 95% CI [0.14, 0.75]) but not symbolic threats (indirect effect = 0.009, 95% CI [−0.02, 0.05]). These findings suggested that women who subjectively appraised realistic threats rather than challenges were more likely to adopt a future-oriented perspective during decision-making (Figure 2).

### 3.3. Discussion

Study 2 examined the causal effects of gender intergroup threats (realistic and symbolic) on intertemporal decision-making among women. Although neither type of threat produced significant group differences in the delay discounting rate, both realistic and symbolic gender threats increased the participants’ consideration of future consequences, suggesting enhanced future-oriented thinking. Mediation analyses further revealed that cognitive appraisal fully explained the association between realistic threats and both the delay discounting rate and future consideration, but not for symbolic threats.

These findings emphasised the importance of cognitive appraisal processes in adaptive responses to gender realistic threats. Symbolic threats, related to values and beliefs, did not produce the same effects on intertemporal decision-making or future-oriented thinking as realistic threats. Building on these results, Study 3 employed an imagination-based priming paradigm and assessed group-level cognitive appraisal in a post hoc manner, further delineating the role of cognitive appraisal in the relationship between gender intergroup threats and intertemporal decision-making.

## 4. Study 3

### 4.1. Methods

#### 4.1.1. Participants

A priori power analysis for a one-way ANOVA was conducted with G*Power 3.1 (Faul et al., 2007), using an effect size estimate of *f* = 0.29 based on previous similar paradigms (Wang & He, 2020; Jiang et al., 2022). The results indicated that a minimum sample size of 120 participants was necessary to achieve 80% statistical power at *α* = 0.05. Consequently, a total of 125 female college students between the ages of 18 and 26 years (*M* = 20.59, *SD* = 1.65) were recruited. Following the exclusion of five participants owing to procedural errors, the final sample comprised 120 participants (*n* = 40 per group).

#### 4.1.2. Procedure and Materials

Participants were randomly assigned to one of three conditions: realistic threat, symbolic threat, or control. The study sequence was as follows: (a) completion of the gender identity scale (Cronbach’s *α* = 0.68) to enhance female identity salience; (b) engagement in gender intergroup threat priming followed by a manipulation check; (c) completion of the cognitive appraisal scale; and (d) administration of an intertemporal decision-making computer task and the Consideration of Future Consequences Scale (Cronbach’s *α* = 0.78). The gender identity, consideration of future consequences, and cognitive appraisal scales were identical to those used in Study 2.

Gender Intergroup Threat Manipulation: An imagination-based priming paradigm was adopted to activate gender intergroup threat perceptions (Xu et al., 2019; Wang & He, 2020; Rios et al., 2018).

- Realistic Threat Condition: Participants read a passage describing real-life gender inequalities and completed three tasks: (1) rated their level of concern about experiencing such situations on a 7-point scale; (2) described, in writing, similar events they had heard of or experienced; and (3) imagined and described a similar future scenario they might face (at least 100 words).
- Symbolic Threat Condition: Procedures mirrored those of the realistic threat condition; however, the materials and reflections focused on gender bias (e.g., devaluation of women’s achievements or stereotypes regarding women’s roles).
- Control Condition: Participants recalled memorable childhood events and imagined possible future lifestyle changes.

Intertemporal decision-making computer task: Intertemporal choice preferences were measured using a computerised task developed by Lv et al. (2023). In each trial, participants chose between a fixed immediate reward (¥20) and a larger delayed reward. The delay intervals for the larger reward were 7, 15, 30, 60, and 120 days, and the amount offered ranged between 10% and 500% greater than the immediate reward (i.e., 10%, 20%, 40%, 80%, 120%, 160%, 240%, 360%, 420%, and 500%). The experiment consisted of four runs, with each participant making 200 decisions. Each trial commenced with a 1000 ms fixation period, followed by a blank screen and presentation of the intertemporal decision. The decision phase was self-paced, after which a randomised intertrial interval and feedback were provided. The main dependent measure was the proportion of decisions favouring the delayed option (i.e., the delay–choice ratio).

### 4.2. Results

#### 4.2.1. Manipulation Check

A one-way analysis of variance (ANOVA) revealed significant differences in self-reported threat-related emotions among the three groups, *F*(2, 117) = 19.05, *p* < 0.001, $\eta_{p}^{2}$ = 0.25, 90% CI [0.11, 0.29]. Post hoc comparisons indicated that both the realistic (*M* = 2.89, *SD* = 1.02, *p* < 0.001) and symbolic threat groups (*M* = 2.92, *SD* = 0.89, *p* < 0.001) reported significantly higher levels of threat emotions than the control group (*M* = 1.82, *SD* = 0.79), demonstrating that both threat manipulations were effective.

#### 4.2.2. Effect of Gender Intergroup Threat on Intertemporal Decision-Making

**Figure 3:**
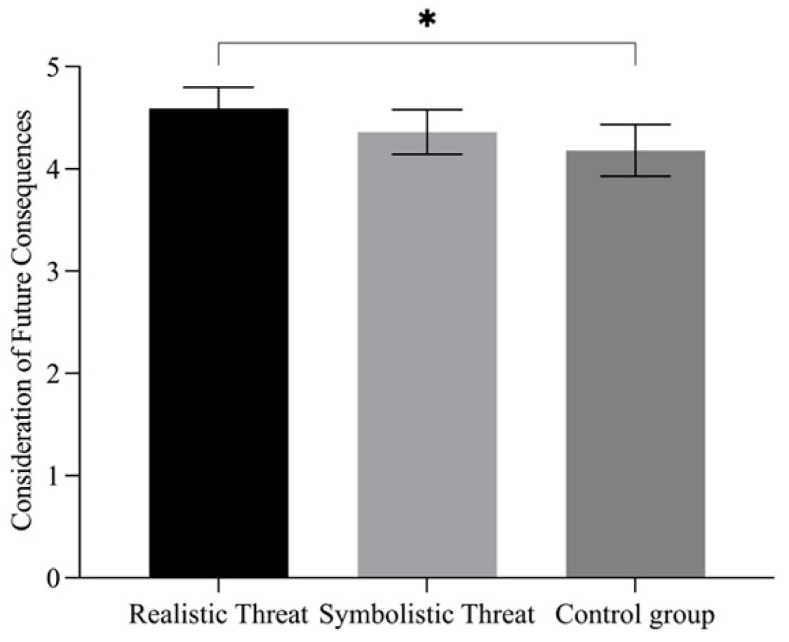
ANOVA results of Consideration of Future Consequences among the Different Intergroup Threat Groups in Study 3. Note. * *p* < 0.05.

Group differences in intertemporal decision-making were examined using an ANOVA. The results revealed no significant differences in the delay–choice ratio across the three groups, *F*(2, 117) = 0.39, *p* = 0.681, $\eta_{p}^{2}$ = 0.007, 90% CI [0.00, 0.03]. However, significant differences were observed in consideration of future consequences (CFC) scores (see Figure 3), *F*(2, 117) = 3.41, *p* = 0.036, $\eta_{p}^{2}$ = 0.06, 90% CI [0.01, 0.10]. Post hoc pairwise comparisons in the same way as for Study 2 indicated that participants in the realistic threat group (*M* = 4.59, *SD* = 0.65) reported significantly higher CFC scores than those in the control group (*M* = 4.18, *SD* = 0.79, *t* = 2.61, *p* = 0.028, Cohen’s *d* = 0.59), whereas the difference between the symbolic threat group (*M* = 4.36, *SD* = 0.68) and the control group was not statistically significant (*t* = 1.13, *p* = 0.497, Cohen’s *d* = 0.25).

#### 4.2.3. Effect of Cognitive Appraisal Style on Intertemporal Decision-Making

**Figure 4:**
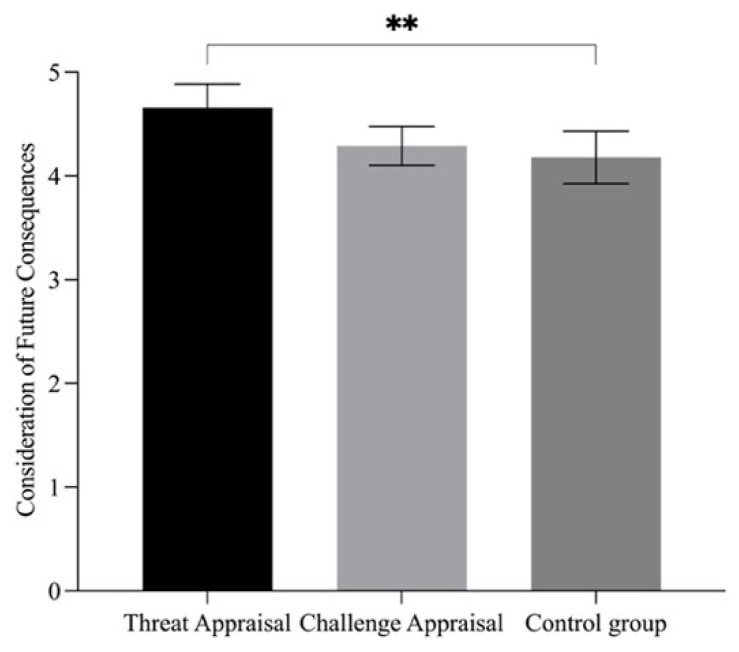
ANOVA results of Consideration of Future Consequences among the Different Appraisal Style Groups in Study 3. Note. ** *p* < 0.01.

To further examine cognitive appraisal’s role, the participants in the realistic and symbolic threat groups were categorised according to appraisal style. Those with a cognitive appraisal score ratio of >1 were assigned to the threat appraisal group (*n* = 38), whereas those with a ratio of ≤1 were assigned to the challenge appraisal group (*n* = 42). The ANOVA indicated no significant group differences in the delay–choice ratio, *F*(2, 117) = 0.40, *p* = 0.67, $\eta_{p}^{2}$ = 0.007, 90% CI [0.00, 0.04]. However, significant group differences were observed for CFC scores (see Figure 4), *F*(2, 117) = 5.11, *p* = 0.007, $\eta_{p}^{2}$ = 0.08, 90% CI [0.01, 0.16]. The post hoc tests revealed that the threat appraisal group (*M* = 4.66, *SD* = 0.73) exhibited significantly higher CFC scores than the control group (*M* = 4.18, *SD* = 0.79, *p* = 0.007), with no significant differences between the challenge appraisal group (*M* = 4.29, *SD* = 0.57) and either the threat appraisal or the control group. These findings suggested that a threat-oriented cognitive appraisal style enhanced individuals’ propensity to consider long-term outcomes.

### 4.3. Discussion

Study 3 employed an imagination paradigm to examine the effects of gender intergroup threats on women’s intertemporal decision-making. Consistent with previous studies, exposure to a realistic gender threat did not significantly influence the behavioural delay–choice ratio compared with the controls. However, experiencing a realistic threat significantly increased the participants’ consideration of future consequences, emphasising enhanced future-oriented thinking. However, symbolic threats did not yield the same effect.

Moreover, post hoc analyses revealed that participants exhibiting a threat-based cognitive appraisal style, regardless of whether they encountered realistic or symbolic threat content, exhibited greater consideration of future consequences than the control group. This finding aligned with Study 2’s mediation results, emphasising the centrality of cognitive appraisal in predicting future-oriented decision-making under a gender threat.

Collectively, these findings suggested that subjective appraisal of gender-related threats amplified future-time orientation among women, potentially facilitating more adaptive decision-making strategies in the context of intergroup threats.

## 5. Discussion

Grounded in intergroup threat theory and cognitive appraisal theory, this study systematically explored the relationship between women’s perceptions of gender intergroup threats and their intertemporal decision-making across three studies, with a particular focus on cognitive appraisal’s mediating role. The findings revealed that stronger perceptions of realistic gender threats were associated with significantly lower delay discounting rates, indicating a greater preference for long-term outcomes. Both realistic and symbolic gender threats enhanced individuals’ consideration of future consequences, with cognitive appraisal fully mediating the link between realistic threats and intertemporal decision-making. Notably, threat appraisal significantly increased women’s tendency to prioritise long-term benefits.

Dissimilar to previous findings, which suggested that stress and threat perceptions often drive individuals toward short-term, impulsive choices (Malesza, 2019; Du et al., 2022), this study’s results indicated that perceptions of gender-based intergroup threats promoted a greater preference for delayed, future-oriented outcomes, particularly under realistic threats. This divergence may reflect the chronic and structural nature of gender inequality, which is persistent, pervasive, and embedded within social systems (Schmader, 2023). Dissimilar to acute stressors, which can be resolved by immediate action, structural gender threats represent slow-moving collective challenges that are not easily managed through short-term responses (Fisseha et al., 2021). Under such chronic threats, women may develop adaptive strategies aimed at securing future benefits, resilience, and resource accumulation, consistent with the slow life history strategy suggested by recent theoretical advances (Gillman et al., 2023). Therefore, adopting a long-term orientation may reflect an adaptive coping mechanism to foster personal development, mitigate ongoing risks, and potentially work toward social change and gender equity (Leavitt et al., 2022; Van Laar et al., 2019; Ryan & Morgenroth, 2024).

Notably, symbolic threats’ influence on temporal preferences appeared to be less robust than that of realistic threats. This difference may be attributable to the distinct nature of these threats: symbolic threats predominantly relate to perceived abstract or distal challenges to group values, norms, or identity and do not directly threaten personal long-term planning or resource allocation, as realistic threats do (Cichocka et al., 2013; C. W. Stephan et al., 2000). Realistic threats typically invoke concerns about concrete losses, such as financial security, personal safety, or status, whereas symbolic threats tend to evoke affective responses related to identity, belonging, or group esteem (W. G. Stephan et al., 2009). Consequently, exposure to symbolic threats is more likely to trigger defensive coping mechanisms, such as group affirmation or heightened in-group solidarity, rather than a recalibration of individual time preferences or self-regulatory behaviour (Jetten et al., 2015).

This study’s results further suggested that although cognitive appraisal did not differentiate the effect of gender threats on women’s intertemporal decision-making, threat appraisal significantly enhanced future orientation. Although cognitive appraisal both buffer and exacerbate the effects of threats, depending on the context (Alter et al., 2010), this study’s findings indicated that, in the face of persistent structural barriers, threat perceptions, along with the appraisal of insufficient resources, motivate a move toward future-oriented coping, delayed gratification, and strategic resource planning (Blascovich & Mendes, 2000; Lazarus & Folkman, 1984; Li & Zhao, 2015). This process may enhance psychological resilience (Guo et al., 2024), identity continuity (Bennamate & Bouazzaoui, 2023), and long-term goal commitment among women in structurally disadvantaged contexts (Alcover et al., 2020; Breakwell, 2021).

### 5.1. Theoretical Contributions and Practical Implications

This study makes important contributions to the literature by elucidating the impact of both group-level factors and cognitive appraisal processes on intertemporal decision-making, thereby enriching intergroup threat theory from a gender perspective. Particularly, this study refined the theoretical framework by revealing how gender-based structural constraints interact with individual cognitive evaluations to shape future orientation. Additionally, this study revealed how women adapt to ongoing gender inequality by emphasising the psychological processes that assist them in balancing immediate challenges with long-term goals in the face of systemic inequality, offering concrete insights into adaptive strategies that mitigate the adverse effects of gender-based structural barriers.

Practically, the results suggest that interventions aimed at empowering women should focus on enhancing cognitive appraisal capabilities and fostering resilience, rather than viewing threat only as a factor promoting negative, short-term outcomes. Organizations, policymakers, and educational institutions can benefit from these insights by designing programs that support long-term planning, resource accumulation, and personal development among women encountering ongoing gender-related threats. For industry, the study underscores the importance of providing supportive environments that recognize and address chronic structural barriers—thereby enabling women to plan for and invest in their futures with greater confidence and stability.

### 5.2. Limitations and Conclusions

Despite these contributions, this study has several limitations, emphasising opportunities for future studies. First, given that female college students may exhibit distinct decision-making tendencies (e.g., heightened future orientation but limited exposure to high-stakes financial trade-offs; Atherton et al., 2021; Reimers et al., 2009), future studies should include diverse samples across ages, occupational backgrounds, and socioeconomic strata to test effect robustness. Additionally, this study focused on intertemporal decision-making, which is conceptually and empirically related to risk decision-making (Zhou et al., 2019). Existing research has established gender differences in risk-related choices (Singh et al., 2020; Villanueva-Moya & Exposito, 2021). Future studies should systematically examine the effects of gender intergroup threats on risk-based and other consequential real-world decisions. Finally, unexamined moderators, such as relationship mobility (Thomson et al., 2018) and feminism identification (Cichocka et al., 2013; Fischer et al., 2000), which may shape adaptive responses, should be incorporated to clarify the boundary conditions of gender intergroup threat effects. In summary, this study advances our understanding of the psychological processes underlying women’s adaptive long-term decision-making under conditions of structural gender threats, emphasising the potential mechanisms for fostering resilience. By addressing these remaining issues, future studies can further elucidate these processes and provide a stronger empirical foundation for interventions and policies dedicated to women’s empowerment and the promotion of gender equity.

## Figures and Tables

**Table 1:** Study 1: Descriptive Statistics and Bivariate Correlations.

Variable	*M*	*SD*	1	2	3	4	5	6	7
1. Age	20.96	2.54	1						
2. Gender Identity	5.43	0.82	0.03	1					
3. Realistic Threat Perception	5.04	1.07	−0.12 *	0.18 **	1				
4. Symbolic Threat Perception	5.25	0.81	−0.06	0.12 *	0.68 ***	1			
5. Delay Discounting Rate (*k*_0_)	−4.18	1.84	0.14 *	−0.02	−0.18 **	−0.06	1		
6. Subjective Social Class	4.74	1.48	0.22 **	−0.03	−0.08	−0.08	0.18 **	1	
7. Objective Social Class	3.99	1.39	−0.09	−0.08	0.03	0.03	0.05	0.31 ***	1

Note. * *p* < 0.05, ** *p* < 0.01, *** *p* < 0.001.

**Table 2:** Hierarchical Regression Results for the Delay Discounting Rate Regressed on the Predictor Variables.

Model	Predictor	*B* (*SE*)	*β*	*t*	*p*	*R* ^2^	∆*R*^2^	*F*
1	Age	0.07 (0.04)	0.10	1.71	0.089	0.04	0.04	6.25
	Subjective social class	0.20 (0.08)	0.16	2.65	0.009			
2	Age	0.06 (0.04)	0.08	1.37	0.171	0.07	0.06	5.44
	Subjective social class	0.19 (0.07)	0.15	2.58	0.010			
	Realistic threat perception	−0.40 (0.14)	−0.23	−2.91	0.004			
	Symbolic threat perception	0.27 (0.18)	0.12	1.49	0.138			

**Table 3:** Mediating Role of Cognitive Appraisal.

	Effect	SE	95% CI
Realistic Threat → Delay Discounting Rate	0.718	0.36	[−0.01, 1.44]
Realistic Threat → Cognitive Appraisal → Delay Discounting Rate	−0.285 *	0.13	[−0.56, −0.05]
Symbolic Threat → Delay Discounting Rate	0.779 *	0.34	[0.11, 1.45]
Symbolic Threat → Cognitive Appraisal → Delay Discounting Rate	−0.022	0.04	[−0.10, 0.07]
Realistic Threat → Consideration of Future Consequences	0.255	0.16	[−0.07, 0.58]
Realistic Threat → Cognitive Appraisal → Consideration of Future Consequences	0.114 *	0.07	[0.14, 0.75]
Symbolic Threat → Consideration of Future Consequences	0.447 *	0.15	[0.01, 0.25]
Symbolic Threat → Cognitive Appraisal → Consideration of Future Consequences	0.009	0.02	[−0.02, 0.05]

Note. * *p* < 0.05.

## Data Availability

The data that support the findings of this study are available from the corresponding author upon reasonable request.
